# Effects of the COVID-19 Mitigation Measures on Alcohol Consumption and Binge Drinking in College Students: A Longitudinal Survey

**DOI:** 10.3390/ijerph18189822

**Published:** 2021-09-17

**Authors:** Margarida Vasconcelos, Alberto Crego, Rui Rodrigues, Natália Almeida-Antunes, Eduardo López-Caneda

**Affiliations:** Psychological Neuroscience Laboratory (PNL), Research Center in Psychology (CIPsi), School of Psychology, University of Minho, 4710-057 Braga, Portugal; alberto.crego@psi.uminho.pt (A.C.); id8512@alunos.uminho.pt (R.R.); id8511@alunos.uminho.pt (N.A.-A.); eduardo.lopez@psi.uminho.pt (E.L.-C.)

**Keywords:** alcohol, binge drinking, COVID-19, pandemic, young adults, college students, alcohol craving, stress, anxiety, depression

## Abstract

To “flatten the curve” of COVID-19 contagion, several countries ordered lockdowns amid the pandemic along with indications on social distancing. These social isolation measures could potentially bring alterations to healthy behavior, including to alcohol consumption. However, there is hardly any scientific evidence of the impact of such measures on alcohol consumption and binge drinking (BD) among young adults, and how they relate to alcohol craving, stress, anxiety, and depression levels. We addressed these questions by conducting a longitudinal study with 146 Portuguese college students—regular binge drinkers (regular BDs), infrequent binge drinkers (infrequent BDs) and non-binge drinkers (non-BDs)—in three moments: before the pandemic (Pre-Lockdown), during lockdown (Lockdown) and 6 months after (Post-Lockdown). Results revealed that regular BDs decreased alcohol use during Lockdown, a change in behavior that was even greater during Post-Lockdown, when regular BDs displayed similar levels of consumption to infrequent/non-BDs. Additionally, alcohol craving and living with friends were predictive of alcohol use during Lockdown, whereas stress, anxiety, and depression symptoms did not contribute to explain changes in drinking behavior. Collectively, the results suggest that BD in young Portuguese college students can be stopped when the contexts in which alcohol intake usually takes place are suppressed, which may have important implications for future prevention and intervention strategies.

## 1. Introduction

In late 2019, the world came to know of a cluster of cases of the Coronavirus disease 2019 (COVID-19) that quickly spread around the globe. After the World Health Organization (WHO) declared the COVID-19 outbreak a pandemic [1], governments around the world were urged to implement serious mitigation measures. Between March and May 2020, the Portuguese government issued several nationwide lockdown directives for a period of six weeks that required citizens to stay home unless conducting “essential activities”, while also defining a ban on the sale of alcoholic beverages after 8 pm in supermarkets. Many concerns were raised in the scientific literature regarding how both individuals and communities would react, not only to the stress inherent to the pandemic context, but to the bulk of mass home confinement directives for long periods of time [2]. WHO inclusively launched a campaign to warn the general public about the potential risks associated with alcohol consumption while social isolation measures prevailed, namely the higher probability of prospective alcohol use disorders [3]. A growing number of scientific works predicted that social distancing and quarantining measures imposed by the outbreak would lead to social isolation and augmented stress, which in turn would drive many to resort to alcohol as a coping mechanism, leading to increases in alcohol-related problems and alcohol abuse disorders, and a potential jump in harmful and heavy drinking [4,5,6,7,8,9,10,11,12]. A recent US survey indicated that 60% of adults increased their alcohol intake during COVID-19, with people suffering more from COVID-19-related stressors having consumed more and for longer periods than others [13]. The problem is that, if indeed consumption rises due to the pandemic, not only will the national disease burdens associated with alcohol be worsened [14,15], but the COVID-19 load will also be heavier due to the damage heavy alcohol intake does to individuals’ immune systems [16].

However, the evidence is still scarce regarding how drinking patterns changed through the COVID-19 confinement periods and afterwards, particularly in young adults. Rehm and collaborators [7] defended that the scenario of increased alcohol consumption due to COVID-19 mitigation measures would be just one of two possibilities; the alternative scenario—and the more plausible one, according to the authors—postulated lower levels of alcohol intake due to a higher difficulty to access it, both physically and financially. The authors suggest that losses in financial capacity and restrictions to access alcoholic beverages (e.g., home confinement, the closing of on-premise consumption sites, bans to alcohol sales, and social distancing) might lead to reductions in the levels of alcohol use in the immediate term [7]. Integrating both approaches, the most comprehensive systematic review to date on the effects of past economic crises on alcohol consumption revealed that budget constraints are associated with decreases in consumption, but also to more harmful drinking, particularly in men, due to psychological distress [17].

The extant research on COVID-19-related changes in alcohol consumption have showed mixed findings across different countries and depending on particular characteristics of the samples under study. Research conducted in the UK, Australia, Germany, and Norway indicated that 21%, 26.6%, 34.7%, and 13%, respectively, of the people who drink alcohol were drinking more during lockdown [18,19,20,21]. Conversely, a very recent large-scale study in 21 countries points to a general decrease in alcohol use (except in Ireland and the UK), motivated by a reduction in the frequency of heavy episodic drinking [22]. Some of these studies revealed that higher consumption was associated with the presence of depressive symptoms [18,19] and with higher levels of perceived stress [20].

Besides understanding the effects of the current pandemic on alcohol consumption, it is important to clarify if BD behavior in young adults has changed and, if so, how. BD is the nomenclature given to the intake of large quantities of alcohol over short periods of time, typically defined as taking five alcoholic beverages (four in women) in a row in two hours [23,24,25,26]. This alcohol consumption pattern is highly prevalent between adolescent and young people in most western countries, including Portugal [27,28,29], and it has become a major public health concern in European countries and the USA involving profound individual and social costs such as violence and vandalism [30,31], risky or forced sexual intercourse [32,33], and lower academic achievements [34]. In addition, the alternation between high intake and withdrawal typically observed in the BD behavior may involve serious deleterious effects on the maturing brains of adolescents, namely neurocognitive anomalies linked to prefrontal dysfunction (e.g., poor inhibitory control and working memory operations [35,36,37,38,39]), somewhat similar to the impairments found in alcohol use disorder [40,41].

The literature reveals that college students’ current living conditions are associated with alcohol consumption, with those living without family obligations (i.e., outside parent’s homes, without children, living alone, with roommates, and in rented accommodation) being more likely to consume alcohol and report BD [42,43]. Such “wet” environments—i.e., friendship networks/affiliations or social and residential surroundings where alcohol is highly accessible and cheap—are considered risk factors for BD [44], and it was precisely those environments that the campus closures and lockdowns imposed by COVID-19 disrupted.

The scarce research on BD changes related to COVID-19 in young adults have shown contradicting results. As such, a recent US study reported 60% of Regular BDs increased alcohol consumption [45], while two other US studies and one Belgian study reported reductions in consumption by nearly 40% [46,47] and 68% [48] of the Regular BDs, respectively. These mixing results on BD behavior draw attention to the need of longitudinal studies about changes in alcohol consumption in young adults with distinct drinking profiles, as a function of the pandemic. Indeed, most previous studies have only used self-report, retrospective measures to evaluate changes in drinking patterns related to the pandemic, also ignoring if changes persisted after restrictions were lifted. Thus, the present study sought to shed new light on the consequences of the COVID-19 pandemic in the alcohol use habits of college students as assessed longitudinally. With that purpose, participants were first evaluated on the Fall of 2019, when there was no foreknowledge of COVID-19 (hereinafter referred to as the “Pre-Lockdown” period), then 15 days after the Government’s shelter-at-home nationwide orders (April–May 2020; “Lockdown”), and finally in October 2020, approximately six months after lockdown was lifted (“Post-Lockdown”). We further investigated how personal characteristics (i.e., gender, age of drinking onset, etc.), as well as situational aspects such as affective mood (i.e., stress, anxiety, and depression levels), craving levels, lifestyle and social contextual factors (e.g., being quarantined, the presence of housemates) contributed to individuals’ engagement in alcohol consumption and BD. Considering both the restrictions on alcohol availability and social gatherings, and the influence of social and environmental factors on BD behavior, we postulated that, in the immediate term (i.e., Lockdown), college students would diminish their alcohol consumption and binge episodes, a reduction we predicted would be overturned by the lifting of restrictions during the Post-Lockdown period. We also hypothesized that COVID-19-related negative affective states, such as stress, anxiety, and depression symptoms, would be associated with increased alcohol consumption, particularly among Regular BD.

## 2. Materials and Methods

### 2.1. Participants and Design

The present study used a longitudinal design to survey a sample of college students during the period of one year. The study encompassed three sequential moments of assessment: Pre-Lockdown (i.e., before the pandemic; Fall 2019), Lockdown (i.e., during the compulsory Portuguese lockdown; April–May 2020) and Post-Lockdown (i.e., six months after lockdown; October–November 2020). We started by recruiting a convenience sample of college students at the University of Minho (Braga, Portugal) in October 2019 with the aim of collecting data for an electroencephalography study investigating the effects of BD on brain activity in young adults. At the time, it was impossible to foresee the occurrence of the COVID-19 pandemic, and therefore the inclusion criteria were limited to being a college student and being under the age of 30. Three hundred and twenty-two participants were recruited and completed a paper and pencil survey in a classroom. After the pandemic started, these individuals were contacted and asked to participate in a follow-up study on their alcohol consumption patterns in the context of the COVID-19 outbreak (i.e., Lockdown assessment; *n* = 146) filling out a confidential, self-administered, online website survey on Qualtrics (https://www.qualtrics.com; accessed on 1 April 2021). In order to explore changes in drinking behavior that had happened as a consequence of the confinement, we decided to only include in the Pre-Lockdown assessment the subjects that had completed the Lockdown evaluation afterwards (i.e., Pre-Lockdown assessment; *n* = 146). After six months from the Lockdown assessment, with the confinement over and the mitigation measures lightened, participants were asked to enroll in the last instance of evaluation by completing a new online survey on the same web platform (i.e., Post-Lockdown assessment; *n* = 93). Each survey took approximately 20–25 min to complete. It is relevant to highlight that the initial purpose of data collection was not to conduct a longitudinal study evaluating the effects of COVID-19 mitigation measures on alcohol consumption; that fact precluded us to recruit a bigger sample of college students at the Pre-Lockdown assessment and to have additional inclusion criteria. Although the data was anonymous, a participant code was created for all students to guarantee the identity of the participants could not be readily determined and to ensure there was no data duplication. Participants gave informed consent for inclusion before the participation in the study and at the start of each survey. The study was conducted in accordance with the Declaration of Helsinki, and received approval by the Institutional Ethics Committee for Social Sciences and Humanities of the University of Minho, Braga, Portugal (CECSH 078/2018).

### 2.2. Measures

The assessment protocol consisted of a battery of questions about socio-demographics, behavior and lifestyle changes associated with the pandemics, and alcohol consumption and craving, and included the Portuguese versions of the Alcohol Use Disorder Identification Test (AUDIT) [49,50], the Penn Alcohol Craving Scale (PACS) [51] and the Depression Anxiety Stress Scales 21 (DASS-21 [52]).

#### 2.2.1. Sociodemographic and Lifestyle Characteristics

The survey contained socio-demographic questions, namely age, gender, educational level, and previous diagnoses of psychopathological conditions. During the Lockdown and Post-Lockdown assessment, the survey was extended to incorporate questions related to lifestyle, namely individuals with whom participants were sharing the house, marital status, use of substances, and questions specific to the pandemic context, namely about adhering to quarantine and being infected.

#### 2.2.2. Assessment of Alcohol Use, Craving, and BD

We used the Portuguese version of the AUDIT [50] (Cronbach’s α = 0.86) as a screening tool for drinking behavior and alcohol-related problems. Particularly, this instrument was used to determine whether the participants fulfilled the BD criteria. Based on the Pre-Lockdown AUDIT total score, and on the score obtained in AUDIT Question 3, the sample was divided into three groups: regular binge drinkers (regular BDs), i.e., those who drink five or more alcoholic beverages in a single occasion at least once a month (AUDIT Q3 ≥ 2; *n* = 59), infrequent binge drinkers (infrequent BDs), i.e., those who drink five or more alcoholic beverages in a single occasion less than once a month (AUDIT Q3 = 1; *n* = 36) and non-binge drinkers (non-BDs), i.e., subjects who never drink five or more alcoholic beverages in a single occasion (AUDIT Q3 = 0 and with a total AUDIT score ≤ 4; *n* = 51). In addition, participants were asked about: (1) the number of alcoholic beverages consumed every day, during a typical week, (2) the age at which they started drinking regularly, and (3) drunkenness (i.e., the percentage of times participants drink alcohol and end up getting drunk). The Portuguese version of the PACS [53] (Cronbach’s α = 0.94) was also used in the Lockdown and Post-Lockdown assessments to measure the level of alcohol during the previous week.

#### 2.2.3. Assessment of Stress, Anxiety, and Depression

We used the DASS-21 [52] which consists of three subscales—Stress, Anxiety, and Depression—assessing symptoms experienced by the individuals in the last week. DASS-21 has been translated into Portuguese [54] and validated for the Portuguese population as Escalas de Ansiedade, Depressão e Stress (EADS-21 [55]; 0.74 < Cronbach’s α < 0.85, for the three sub-scales).

### 2.3. Analyses

The total number of drinks per week was used as an index of ‘alcohol consumption’ at the three moments. In order to examine the percentage of participants that increased, decreased, or kept the same alcohol consumption between moments, we computed differences between each moment (i.e., Pre-Lockdown minus Lockdown; Pre-Lockdown minus Post-Lockdown; and Lockdown minus Post-Lockdown). Linear regressions were implemented to identify the significant predictors of alcohol consumption at Pre-Lockdown, Lockdown, and Post-Lockdown, namely: gender, age, drinking onset, drunkenness, alcohol craving, quarantining, housemates, and stress, anxiety, and depression levels. Dummy variables were created for the variables gender, housemates (i.e., living with family, with friends and alone) and quarantine. A paired samples *t*-test was performed to compare alcohol craving levels between Lockdown and Post-Lockdown. Statistical analyses were performed in IBM^®^ SPSS^®^ Statistics (Release 27.0.0). For all analyses, *p*-values lower than 0.05 were considered statistically significant. The graphic representation of the regression models was created with R software version 3.6.1 [56] using the ggplot2 function [57].

To examine the relationship between drinking behaviour and moments of assessment (i.e., Pre-Lockdown, Lockdown, and Post-Lockdown) and group (i.e., Non-BDs, Infrequent BDs, and Regular BDs) and an interaction term between moment of assessment and group (i.e., Moment × Group), linear mixed-effects models (LMMs) were estimated. LMMs are particularly appropriate for the analysis of nested structured data, as is the case of this study, using the restricted maximum likelihood estimation and are well-known for being robust to missing values [58]. We used the total number of drinks consumed over a typical week as outcome measures in the LMM. Additional LMMs were calculated to investigate the relationship between affective states (i.e., stress, anxiety, and depression levels) and the moments of assessment (i.e., Lockdown and Post-Lockdown). Additionally, we calculated three independent LMMs to investigate if stress, anxiety, and depression interacted with moment of assessment and drinking group to explain alcohol consumption. As the data were nested within participants, participants were treated as random effects. Statistical analyses for linear mixed effects models were carried out in R (R Core Team, 2017), using lme4 package and the lmer function. *p*-values for pairwise comparisons were adjusted using the Bonferroni method (alpha level < 0.05).

## 3. Results

### 3.1. Relationship between Socio-Demographic/Lifestyle Variables and Alcohol Consumption before, during, and after COVID-19 Lockdown

Survey demographics and the lifestyle characteristics of the sample, as well as information regarding COVID-19 infection are presented in Table 1.

Parameter estimates and *p*-values for each regression model, across moments of assessment, are presented in Table 2. On average, during Post-Lockdown, male college students showed an alcohol consumption level 1.31 points higher than that of female students (*R*^2^ = 0.05; *R*^2^_adj_ = 0.04; *p* = 0.040; β = 1.31). The older participants were, the greater their consumption during Post-Lockdown (*R*^2^ = 0.09; *R*^2^_adj_ = 0.08; *p* = 0.003; β = 0.46). The earlier the age at which participants started drinking regularly, the higher the alcohol consumption before the pandemic (*R*^2^ = 0.05; *R*^2^_adj_ = 0.04; *p* = 0.015; β = −1.29). A higher percentage of past drunkenness was associated with higher consumption during both Pre-Lockdown (*R*^2^ = 0.24; *R*^2^_adj_ = 0.23; *p* < 0.001; β = 0.11) and Lockdown (*R*^2^ = 0.04; *R*^2^_adj_ = 0.03; *p* = 0.023; β = 0.03), whereas increased levels of craving were associated with higher alcohol consumption (i.e., drinks per week) during Lockdown (*R*^2^ = 0.27; *R*^2^_adj_ = 0.26; *p* < 0.001; β = 1.27) and Post-Lockdown (*R*^2^ = 0.07; *R*^2^_adj_ = 0.06; *p* = 0.010; β = 0.22). Likewise, sharing the house with friends (as compared with living with family or alone) was associated with increased drinking during Lockdown (*R*^2^ = 0.05; *R*^2^_adj_ = 0.04; *p* = 0.020; β = 4.49), while living with family during Lockdown (as compared with living with friends or alone) was associated with lower levels of drinking (*R*^2^ = 0.04; *R*^2^_adj_ = 0.03; *p* = 0.039; β = −3.76).

### 3.2. Changes in Alcohol Consumption before, during, and after COVID-19 Lockdown

During Lockdown, 48% of participants decreased their alcohol intake, while 15% increased, and 37% did not modify their alcohol use. During Post-Lockdown (as compared to Pre-Lockdown) a similar pattern was found: 57% of participants decreased their alcohol intake, while 9% increased, and 34% drank the same. When Lockdown and Post-Lockdown moments were compared, we found that the majority of respondents kept drinking the same (51%), while 28% decreased consumption, and 21% increased it. When assessing if the levels of craving changed during the pandemic, we found that alcohol craving increased from Lockdown (*M* = 0.9) to Post-Lockdown (*M* = 1.8; *t* (88) = −2.56, *p* = 0.012).

### 3.3. Regular BDs, Infrequent BDs, and Non-BDs’ Changes in Alcohol Consumption before, during, and after the COVID-19 Lockdown

**Figure 1:**
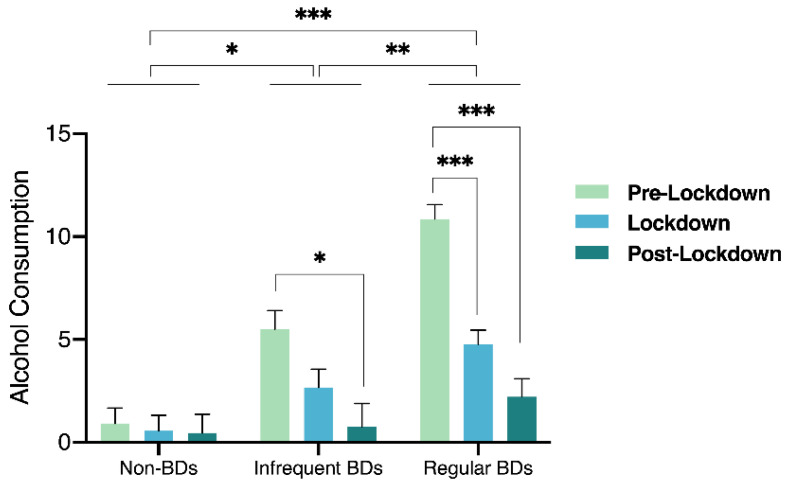
Bar chart depicting the results of the linear mixed-effects model (LMM) testing the associations between the moment of assessment (Pre-Lockdown, Lockdown, and Post-Lockdown) and Group (Non-BDs, Infrequent BDs, and Regular BDs) with alcohol consumption (i.e., drinks per week) and an interaction term (Moment × Group). Asterisks denote significant pairwise comparisons; * *p* < 0.05, ** *p* < 0.005, and *** *p* < 0.001. BD = binge drinkers.

We estimated an LMM with random intercepts (i.e., subject) for testing the associations between the moment of assessment and group of alcohol consumption (i.e., drinks per week) and an interaction term between moment of assessment and group (i.e., Moment × Group). The relation between variables as well as the *p*-values for pairwise comparisons are illustrated in Figure 1.

Moment was not significantly associated with alcohol intake (β = −0.25, *p* = 0.601). However, we found a positive association between Group and alcohol consumption (β = 6.86, *p* < 0.001) and a negative association between the interaction term Moment × Group and alcohol consumption (β = −2.14, *p* < 0.001). As expected, the pairwise comparisons revealed that regular BDs (*M* = 5.9) drank significantly more than infrequent BDs (*M* = 2.9; *p* = 0.002) and non-BDs (*p* < 0.001), and that infrequent BDs drank, in turn, more than non-BDs (*M* = 0.7; *p* = 0.026). Non-BDs’ alcohol consumption did not change across moments (*p* > 0.05 for all comparisons). On the contrary, regular BDs’ alcohol intake diminished from Pre-Lockdown (*M* = 10.9) to Lockdown (*M* = 4.8; *p* < 0.001) and to Post-Lockdown (*M* = 2.2; *p* < 0.001), not differing significantly between Lockdown and Post-Lockdown (*p* = 0.215). Somewhat similarly, infrequent BDs drank less in the Post-Lockdown (*M* = 0.8) comparatively with the Pre-Lockdown period (*M* = 5.5; *p* = 0.010). While, during Pre-Lockdown, the groups differed significantly regarding the number of drinks consumed per week (non-BDs vs. infrequent BDs: *p* = 0.003; non-BDs vs. regular BDs: *p* < 0.001; regular BDs vs. infrequent BDs: *p* < 0.001), during the Lockdown period only the non-BD and the regular BD groups displayed significant differences (*p* = 0.002). In addition, during Post-Lockdown, there were no differences at all between groups. Importantly, regular and infrequent BDs diminished their alcohol consumption from Pre-lockdown to Lockdown and to Post-lockdown, and stabilized their consumption over the course of the pandemic. The variance in alcohol consumption explained by the moment and group variables and their interaction was of 30%, as indicated by the marginal *R*^2^ statistic.

### 3.4. Association between Stress, Anxiety, and Depression, and Alcohol Consumption for Regular BDs, Infrequent BDs, and Non-BDs during the Outbreak

When investigating differences in affective states as a function of the pandemic, we found that moment of assessment was associated with stress (β = 3.18, *p* < 0.001), anxiety (β = 2.36, *p* < 0.001), and depression (β = 2.55, *p* < 0.001), with levels of all three affective states being higher during the Post-Lockdown period as compared with Lockdown. Additionally, there were no significant interactions between stress, anxiety, and depression and moment of assessment and drinking group that could contribute to explain alcohol consumption (*p* > 0.05).

## 4. Discussion

To the best of our knowledge, the present study represents the first longitudinal perspective on alcohol consumption and BD before and during the COVID-19 outbreak in college students. Our findings revealed that the COVID-19 mitigation measures led to significant changes in alcohol intake. In general, students decreased consumption during Lockdown and Post-Lockdown in comparison with the pre-COVID-19 period, and stabilized in a low level of alcohol intake during the pandemic, despite an increase in alcohol craving in Post-Lockdown. Regular BDs diminished their alcohol consumption and continued to drink significantly less than usual, even when there were no more isolation restrictions in effect. Individuals with an infrequent BD behavior also lowered their drinking intake in the Post-Lockdown period. Moreover, we found that having a history of drunkenness, higher craving, and having friends as co-living partners were factors positively associated with alcohol consumption during Lockdown. However, stress, anxiety, and depression symptoms did not contribute to explain changes in drinking behavior during the pandemic in this population.

The findings of the present study lend empirical support to the assumption that measures entailed to mitigate the COVID-19 outbreak strongly affected alcohol consumption, revealing that, in the particular case of Portuguese college students, the Lockdown led to a significant decrease in consumption and to a reduction in risky drinking behavior. Nearly half of our sample decreased alcohol intake due to Lockdown, and an analogous percentage of individuals kept drinking the reduced number of alcoholic beverages per week six months later, when there was no shelter-at-home duty. In fact, during this phase, almost a third of students were drinking even less than before. Most importantly, this study showed a strong change in the consumption behavior of students that regularly practiced BD as the pandemic unfolded in time, indicating a significant decrease in alcohol intake from the pre-pandemic time to Lockdown and Post-Lockdown. These findings agree with recent research conducted worldwide with college students suggesting that COVID-19 lockdown measures, which imposed campus closures and limitations of social contact, led to significant decreases in alcohol consumption in BDs, thus appearing to work as a short-term ecological intervention on BD behavior for these students [22,46,47,59].

Individuals engaging in BD are typically less motivated to change their behaviors due to adjustment to the negative outcomes associated with repeated intoxications [60,61], which makes the design of intervention strategies hard to accomplish. The evidence gathered here supports the idea that BD is a context-related habit, much interconnected with social and enhancement motifs [62,63], and that in face of people’s resistance to change, heavy drinking can be stopped if the context eliciting it is disrupted. The nationwide lockdown and related measures rendered it impracticable to attend social events (e.g., concerts, parties, and celebrations), to meet in-person with drinking peers, and to buy alcoholic beverages at certain periods of the day. It also closed on-premise consumption sites (i.e., bars, restaurants), which created barriers to access alcohol per se but also alcohol-related contexts. Additionally, the fact people were facing a time of great economic instability, in which many families were struggling with financial problems, might have contributed to reduce the buying of alcohol and, therefore, kept college students away from its consumption. This evidence seems to validate Rehm et al.’s predictions regarding the impact of the availability–affordability mechanism during the COVID-19 crisis [7]. At the same time, most students were gathered at home with their families during the Lockdown, which was shown to be a protective factor for alcohol consumption. For college students, being home with family meant recovering parental supervision and control, and being apart from ‘wet’ environments (i.e., sharing houses with friends and peers; fraternities; gatherings at/outside the university campuses) that typically predict risky drinking behavior [42,43,44,64,65,66].

Our findings oppose some recent research on this topic that suggests alcohol consumption and risky drinking patterns increased during the pandemic [20,21,67,68,69,70], as well as previous studies reporting higher consumption during lockdown in individuals with previous history of intense drinking [69,70], but see Kilian et al. [22] for a large-scale study with a contradicting view. It is important to highlight that: a) these studies used miscellaneous samples that substantially differed between each other, and particularly from our sample of college students; and b) the conceptualization of binge/heavy drinking varied considerably between studies, with the majority not adopting a classification that could group respondents as regular BDs. A closer look at the literature reveals that the only study addressing similar research questions in college students found alcohol intake to have diminished over the term of COVID-19 mitigation measures [48]. In fact, the cross-sectional European Alcohol and COVID-19 survey conducted by Killian et al. [22] revealed an overall decrease in alcohol use, mostly driven by less frequent heavy episodic drinking. Additionally, the longitudinal nature of the present study is advantageous in face of previous studies, as they stem from retrospective assessments of behaviors prior to COVID-19 which could have been affected by memory biases.

It is also relevant to reflect upon the evidence of different changes in alcohol consumption during the outbreak as a function of age, namely, to look at young adults. Some studies indicated younger adults (typically aged between 18–39 years) to have higher odds of augmented consumption during the outbreak [21,67] while others suggest people reporting reduced alcohol consumption after lockdown are significantly younger than those reporting the opposite pattern [69]. In their large population-based study, Alpers and colleagues [21] found people belonging to the youngest age groups reported the highest increase in alcohol use during lockdown, which the authors explain with the vulnerability this group faces in terms of financial dependence and economic uncertainty. These findings are dissonant from ours, as we showed reduced alcohol consumption during lockdown in young college students, an age group somewhat similar to Alpers et al.’s youngest subjects. If reframed, these apparent contradicting results are in fact informative of how lockdown distinctively impacted young adults’ drinking habits depending on their personal circumstances, which might vary greatly depending on factors such as potential job loss and family burdens which are absent in most college students. Therefore, this study highlights that the COVID-19 outbreak might have a different impact in alcohol use in different age ranges, particularly in young adults that are college students comparatively with the general population. As such, our findings suggest that COVID-19 mitigation and social distancing measures could have restrained young adults’ ability to maintain the usual social contexts in which most episodes of alcohol consumption and BD occur, which might have led to lower alcohol intake.

The results also suggest that daily social factors and drinking motives might play a more important role than affecting drinking behaviors, which is in line with previous research [71]. We found that students’ stress, anxiety, and depression levels were higher during the Post-Lockdown as compared with Lockdown, which agrees with evidence suggesting increased psychological distress in the general population [72,73] but also in college students during outbreaks of infectious diseases [74,75,76]. Though young adults are less likely to suffer acute symptoms of COVID-19 relative to older individuals, the evidence gathered here reveals how they were nevertheless psychologically impacted by the pandemic. However, stress, anxiety, and depression levels were not associated with alcohol consumption, nor did they moderate the association between group/moment of assessment and drinking, not contributing to explain drinking changes, either over time or as a function of the alcohol consumption profiles of the students.

Regarding sociodemographic factors, we found no significant role of gender in alcohol use before the pandemic and during Lockdown, but being a male was associated with higher intake during the Post-Lockdown, which is in line with the extant research revealing that men usually consume more heavily than women [77,78,79,80]. In addition, living with family seems to have worked as a protective factor for drinking during Lockdown, while sharing the house with friends was associated with higher alcohol use during this period. Even with the restrictions on movement and the low availability of on-premise consumption sites, students confined with friends, possibly also drinking mates, might have had a social context at home that triggered increased off-premise consumption (note that grocery shops remained open).

Finally, the present study presents some limitations that need to be acknowledged. First of all, there are constrains related to the use of self-report measures, namely recall biases, which can sometimes lead individuals to under/overestimate their own behavior. Additionally, the relatively small sample size limits the scope of the study’s findings, and therefore future works using bigger samples are needed to further investigate the research questions addressed here. Additionally, certain factors, such as alcohol craving, and stress, anxiety, and depression levels, were only measured during the pandemic (Lockdown and Post-Lockdown), not allowing for a true pre–post lockdown situation comparison. A gender imbalance was also present in our sample (nearly 8 out of 10 participants were female) meaning that selection bias cannot be ruled out, and that aspect should be considered. It is also important to interpret the current results with caution as they were obtained with a convenience sample of Portuguese-speaking college students and may not generalize to other non-college students’ populations and other contexts or countries. Finally, the third moment of assessment (i.e., Post-Lockdown) was conducted during the fall of 2020 at a time when lockdown was over and most restrictions had been lifted, but when there were still constraints, and the 2nd wave of the epidemic was prevalent. Those unusual conditions might have constrained people to return to their normal routine of social interaction, which made the context of the third assessment different from the baseline assessment (i.e., Pre-Lockdown) and not strictly comparable.

Future studies should also consider including socio-economic background, financial distress, and monetary income as predictor variables of drinking behavior, given the evidence of their role in alcohol consumption (see [22] for a recent evidence). Additionally, it would have been relevant to test the predictive role of pre-lockdown drinking motives (enhancement, social, and coping motives) on consumption; for instance, using the Drinking Motive Questionnaire Revised [81], and further investigating whether coping motives increased during the pandemic and if they were associated to increases in consumption, as recent studies suggest [82,83]. When, in the near future, COVID-19 is no longer a global threat to public health due to massive population vaccination, and there are no remaining restrictions, further studies are needed to evaluate if college students’ drinking patterns and BD behavior returned to baseline levels, or remained lower than initially measured. Finally, as the evidence suggests (1) BD in college students to be an eminently social behavior, and (2) the lower alcohol use to be the result of limitations on social events/interactions plus potential barriers to physically/economically access alcohol, it is likely that college students’ drinking behavior returns to baseline if no new public health policies target this population.

## 5. Conclusions

The present work responded to the current need of gathering longitudinal evidence about possible changes in alcohol consumption and BD behavior among college students stemming from the COVID-19 outbreak. In this vein, the present results revealed that Portuguese college students with a regular BD pattern decreased alcohol use during Lockdown (Spring 2020), a change in behavior that was even greater during Post-Lockdown (Fall 2020). The fact that the BD behavior was reduced/removed, despite the absence of the isolation measures imposed during Lockdown, seems to highlight the social nature of BD in college students, as the disruption of the typical interpersonal contexts where BD occurs (e.g., social meetings, concerts, and celebrations) played a major role in the suppression of such behavior. Additionally, our findings also showed that factors such as craving alcohol and living with friends—as opposed to living with family—were positively associated with alcohol use during Lockdown, whereas stress, anxiety, and depression symptoms did not contribute to explain changes in drinking behavior during the pandemic in this population. Despite the lack of an adequate gender balance in the present study, and although these results are not applicable to all young people but specifically to Portuguese college students, they suggest that the BD behavior in college students—which is the population among which BD is more prevalent and problematic worldwide [49,84,85,86]—can be curbed when the contexts in which alcohol intake typically takes place are suppressed. These findings may have important implications for future prevention and intervention strategies.

## Figures and Tables

**Table 1:** Socio-demographic and lifestyle characteristics of the sample.

Characteristics	Pre-Lockdown		Lockdown (%)	Post-Lockdown (%)
	Percentage	Range		
Gender (% Female|% Male)	81|19			
Age (years) ^1^	19.5 ± 1.5	17–26		
Onset of drinking (years age) ^1^	16.4 ± 1.4	10–21		
Drunkenness *	30 ± 32			
Occupation				
College	97			
College & Work	3			
Education level				
Undergraduate	92			
Bachelor’s degree	7			
Master’s degree	1			
Psychological disorder	3			
COVID-19 infection			1	2
Housemates				
With family			93	84
With friends			6	15
Alone			1	1

Note. Percentages are rounded to units. ^1^ Data correspond to the mean value ± standard deviation. * Percentage of times participants got drunk when drinking.

**Table 2:** Results of the linear regression models.

Variable	Alcohol Consumption								
	Pre-Lockdown			Lockdown			Post-Lockdown		
	β	SE	*p-*Value	β	SE	*p-*Value	β	SE	*p-*Value
Gender ^1^	2.63	1.58	0.098	0.34	1.27	0.791	**1.31**	**0.63**	**0.040**
Age	0.21	0.44	0.638	0.23	0.34	0.493	**0.46**	**0.15**	**0.003**
Drinking onset	**−1.21**	**0.49**	**0.015**	−0.50	0.38	0.193	−0.27	0.22	0.235
Drunkenness	**0.11**	**0.02**	**<0.001**	**0.03**	**0.01**	**0.023**	0.01	0.01	0.171
Stress	-	-	-	−0.03	0.16	0.830	−0.00	0.05	0.970
Depression	-	-	-	0.04	0.18	0.805	0.01	0.05	0.817
Anxiety	-	-	-	−0.09	0.25	0.713	−0.01	0.05	0.751
Alcohol Craving	-	-	-	**1.27**	**0.79**	**<0.001**	**0.22**	**0.08**	**0.010**
Quarantine	-	-	-	−0.92	1.74	0.600	1.05	0.89	0.241
Living with family	-	-	-	**−3.76**	**1.79**	**0.039**	−0.90	0.69	0.200
Living with friends	-	-	-	**4.49**	**1.90**	**0.020**	1.06	0.72	0.142
Living alone	-	-	-	−1.53	5.02	0.761	−1.18	2.44	0.630

Note. Values result from bivariate regression analyses. β = unstandardized regression coefficient; SE = standard error; ^1^ Male gender; Statistically significant models are highlighted in bold.

## Data Availability

The data presented in this study are openly available in FigShare at 10.6084/m9.figshare.14755278 (accessed on 22 August 2021).
